# Supplementary material for: Increased Precipitation Shapes Relationship between Biochemical and Functional Traits of Stipa glareosa in Grass-Dominated Rather than Shrub-Dominated Community in a Desert Steppe
Source: Plants (Basel). 2020 Oct 29;9(11):1463. doi: 10.3390/plants9111463 (PMC7692965; doi:10.3390/plants9111463)
Supplement: Supplementary file 1 [file plants-09-01463-s001.pdf]

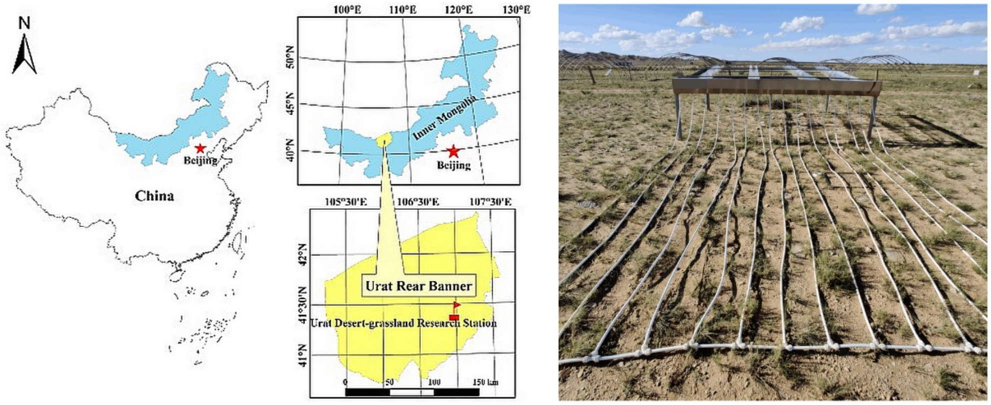

Figure S1 Location of the study area and long-term study site for manipulated precipitation treatments.

Table S1. Community structural characteristics of grass and shrub communities

Precipitation	Above Biomass (g/m^2)		Total Coverage (%)		Species richness		Density	
	Grass	Shrub	Grass	Shrub	Grass	Shrub	Grass	Shrub
CK	13.20±3.90	52.85±17.07	31.67±1.05	31.83±2.24	9.50±0.85	9.17±1.22	65.81±9.33	38.01±6.64
+20%	16.36±3.14	49.58±12.30	33.5±1.52	35.33±2.11	13.00±1.37	12.50±0.89	73.15±2.43	52.20±8.50
+40%	21.44±5.22	63.43±23.63	41.67±1.05	42.50±2.28	14.83±0.70	13.67±0.56	62.65±6.78	58.81±9.23
+60%	20.67±3.74	83.54±13.67	46.67±1.05	46.33±3.23	14.33±1.12	13.00±0.68	72.85±9.40	54.39±9.22

Table S2. Actual precipitation of each treatment in growth season in 2018

Treatments	Actual precipitation (mm)					
	May	Jun	Jul	Aug	Sep	Growth season
CK	8.0	10.0	73.2	125.4	30.4	247
+20%	9.6	12.0	87.8	150.5	36.5	296.4
+40%	11.2	14.0	102.5	175.6	42.6	345.9
+60%	12.8	16.0	117.1	200.6	48.6	395.1

Table S3. Description of measured functional and biochemical traits

Traits	Description
Plant height	Competition ability of plant space resources
Leaf thickness	Physical support capacity and water storage ability; light interception
SLA	Plant growth and survival strategies; ability of plants to acquire resources
LDMC	Tolerance of plants and ability to acquire resources, which generally negatively related to SLA
LCC	Ecological strategy and primary productivity of plants
LNC	Absorption characteristics of soil nutrients; water use efficiency; photosynthetic rate of leaves
Ca	Absorption of light energy; conversion of light energy
Cb	Absorption of light energy
Cx	Chloroplast protection
Proline	Response to environmental stress; maintenance of homeostasis in cells
protein	Osmotic adjustment
REC	Cell membrane permeability
SOD	Scavenging of reactive oxygen species
POD	Scavenging of reactive oxygen species
MDA	Membrane lipid peroxidation production

Table S4. The *F* values of two way-ANOVA for effects of precipitation, community and their interactions on biochemical and functional traits of *Stipa glareosa*

Variables	Precipitation	Community	Precipitation×Community
Plant height	2.912*	0.041	1.216
Leaf thickness	0.758	0.164	2.318*
SLA	4.163*	8.002**	1.350
LDMC	4.873**	15.815***	1.444
LCC	2.296	11.299**	2.833*
LNC	1.189	3.876	1.089
Ca	0.541	206.870***	2.542*
Cb	5.622**	144.822***	0.615
Cx	0.120	180.278***	2.291*
Proline	2.721	2.643	4.169**
protein	5.652**	30.278***	3.775**
REC	13.357***	23.209***	1.015
SOD	15.396***	194.132***	0.750
POD	5.653**	18.398***	0.721
MDA	3.891*	33.151***	2.128

*: $P<0.05$, **: $P<0.01$, ***: $P<0.001$

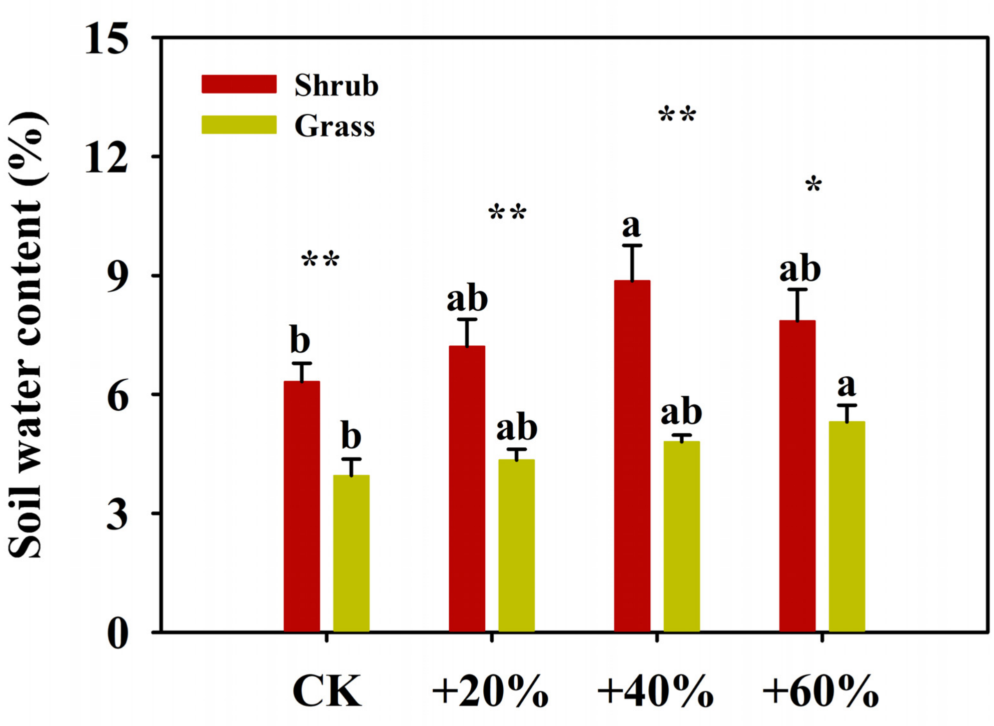

Figure S2. Effects of increased precipitation on soil water content in shrub and grass community in the desert steppe.

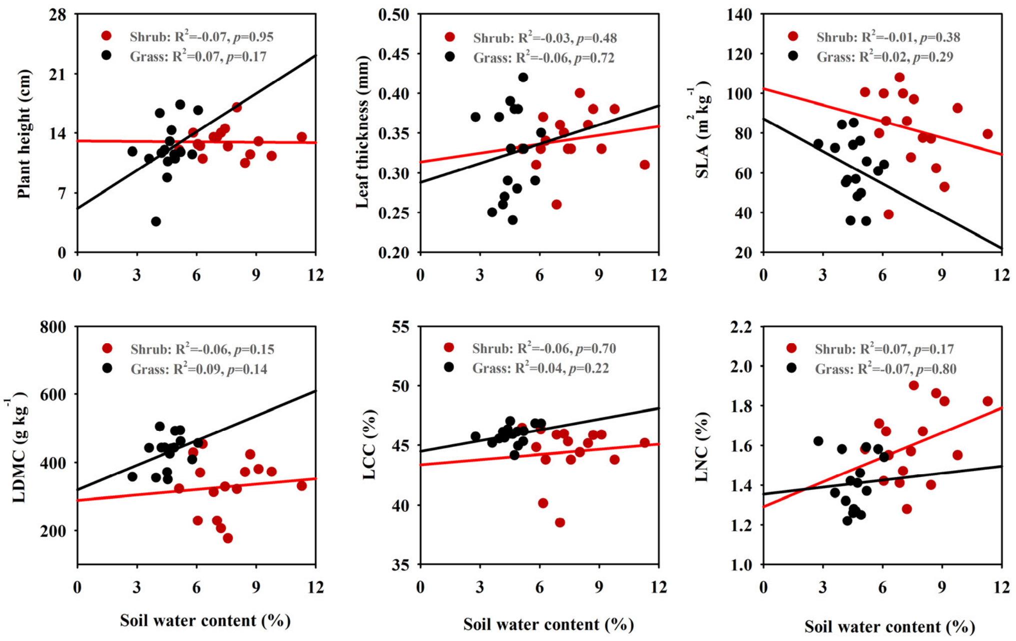

Figure S3. Correlations between soil water content and functional traits of *Stipa glareosa* in the shrub- and grass-dominated communities. SLA, specific leaf area; LDMC, leaf dry matter content; LCC, leaf carbon content; LNC, leaf nitrogen content.
